# Formulation and Bioequivalence Evaluation of a Miniaturized Fexofenadine Hydrochloride Tablet

**DOI:** 10.3390/pharmaceutics17060756

**Published:** 2025-06-08

**Authors:** Woo-Yul Song, Chang-Soo Han, Won-Sang Yu, Jae-Won Jang, Gyoung-Won Kim, Yoo-Shin Jeon, Young-Jin Kim, So-Jeong Jeong, Ji-Hyun Kang, Dong-Wook Kim, Yun-Sang Park, Chun-Woong Park

**Affiliations:** 1 Department of Pharmacy, Chungbuk National University, Cheongju 28644, Republic of Korea; wysong@yuyu.co.kr (W.-Y.S.); hsoo0805@naver.com (C.-S.H.); 7777ytrewq@gmail.com (Y.-J.K.); thwjd07@gmail.com (S.-J.J.); 2 Yuyu Pharma, Inc., Seoul 04598, Republic of Koreaysjeon@yuyu.co.kr (Y.-S.J.); 3 Department of Pharmacy, Jeonbuk National University, Jeonju 54896, Republic of Korea; jhkanga@jbnu.ac.kr; 4 Department of Pharmacy, Wonkwang University, Iksan-si 54670, Republic of Korea; pharmengin1@wku.ac.kr; 5 P2KBio, Cheongju 28160, Republic of Korea; parkys@p2kbio.co.kr

**Keywords:** miniaturized, organic solvent, porous carrier, scale-up, bioequivalence

## Abstract

**Background:** Fexofenadine hydrochloride (FEX) is widely used to treat allergic rhinitis. However, poor solubility, high cohesiveness, and risk of polymorphic transformation present significant formulation challenges. Conventional FEX tablet formulations are large and may pose swallowing difficulties for patients with dysphagia. Therefore, a miniaturized FEX tablet that maintained bioequivalence with the marketed product was developed. **Methods:** An organic solvent-based binder and porous carrier enhanced solubility, flowability, and process efficiency. The formulation was optimized using a design of experiments approach to assess the effects of tablet size and porous carrier incorporation on dissolution and residual solvent content. Scale-up feasibility was evaluated using Froude number-based process optimization, and stability studies were conducted under accelerated conditions (40 °C and 75% relative humidity) to ensure long-term formulation robustness. **Results:** The miniaturized tablet exhibited dissolution at pH 4.0 and pH 6.8 equivalent to that of the reference product, whereas a faster dissolution rate was observed at pH 1.2. No significant changes were observed in the dissolution rate, crystalline structure, or impurity levels over six months. An in vivo bioequivalence study demonstrated that the test formulation met the bioequivalence criteria, with 90% confidence intervals for the area under the curve and the C_max_ falling within the regulatory acceptance range. **Conclusions:** A miniaturized and commercially viable fexofenadine hydrochloride tablet was developed (44% weight reduction and 50% volume reduction compared to the marketed product). The organic solvent-based binder and porous carrier system improved manufacturing efficiency, stability, and solubility, thereby ensuring compliance with regulatory standards. These findings provide valuable insights into size reduction, solubility enhancement, and large-scale production strategies for the development of future pharmaceutical formulations.

## 1. Introduction

Allergic rhinitis, also known as hay fever, is characterized by symptoms such as frequent sneezing, nasal congestion, nasal itching, and persistent rhinorrhea, all of which can significantly affect the daily activities and quality of life of affected patients [1]. It is a major public health concern affecting approximately 400 million people worldwide, and its prevalence continues to increase every year, making it a global issue [2,3]. Allergic rhinitis symptoms are primarily caused by histamine acting on histamine 1 (H_1_) receptors. During the early IgE-dependent allergic response, histamine, along with other mediators, increases the permeability of capillaries and arterioles, leading to enhanced vascular permeability to plasma proteins. Furthermore, histamine induces smooth muscle contraction in the bronchi and stimulates mucus secretion in the nasal glands, ultimately contributing to the characteristic allergic rhinitis symptoms [4].

Antihistamines and corticosteroids are commonly used pharmacological treatments for allergic rhinitis. According to clinical practice guidelines in the United States, oral antihistamines are recommended as the first-line treatment for allergic rhinitis [5,6]. Fexofenadine hydrochloride (FEX) is the active metabolite of terfenadine. Unlike first-generation antihistamines, it does not cross the blood–brain barrier, thereby minimizing sedation-related side effects and reducing disruption to daily activities [7]. Furthermore, its extended half-life enables less frequent dosing, while maintaining therapeutic efficacy. FEX-based products have gained significant popularity in the global market [8]. However, owing to the physicochemical properties of FEX, conventional tablet formulations are relatively large, with a major axis of approximately 17.4 mm, posing challenges for oral administration, particularly for patients with swallowing difficulties. According to the FDA’s guidance titled “Size, Shape, and Other Physical Attributes of Generic Tablets and Capsules”, the single dimension of a generic tablet should generally not exceed 17 mm. However, since the reference listed drug (RLD) for fexofenadine has a maximum dimension of 17.4 mm, matching or reducing the tablet size relative to the RLD is still considered important for ensuring patient acceptability and compliance [9]. In this study, a tablet formulation smaller than 17 mm was designed to improve patient acceptability and align with FDA recommendations.

As a Biopharmaceutics Classification System (BCS) Class II drug, FEX is characterized by low water solubility and high permeability. Its aqueous solubility is relatively low, with a concentration of approximately 0.056 mg/mL at 25 °C, while demonstrating moderate solubility under acidic and basic conditions, reaching 0.205 mg/mL in 0.1 N HCl and 0.184 mg/mL in pH 6.8 buffer [10]. The drug demonstrates strong cohesive interactions, leading to particle agglomeration when the excipient levels are insufficient, which may hinder dissolution. In addition, because of the polymorphic nature of FEX, phase transformations have been observed during manufacturing, particularly during granulation in the presence of water. FEX exists in three distinct solid-state forms: Forms I, II, and III, each of which exhibits unique physicochemical properties [11]. Among these, only Form I is used as a commercially available active pharmaceutical ingredient (API) [12]. However, during manufacturing, Form I can transition into Form II, which can significantly affect granule flowability, density, dissolution rate, and drug release characteristics [13]. To develop a miniaturized formulation of FEX, it is crucial to design an optimized formulation that maintains the drug dissolution while minimizing the amount of excipients. Additionally, for commercial feasibility, process optimization studies must be conducted to control crystallinity and dissolution behavior, followed by a comprehensive stability evaluation to ensure formulation robustness.

Organic solvent-based granulating liquids offer numerous advantages in the pharmaceutical and material sciences. These binders play critical roles in improving dispersion, preventing ingredient agglomeration, and maintaining the crystalline stability of APIs [14,15]. Their versatility makes them invaluable for the development of drug formulations, solid dispersions, coating technologies, and nanomaterial stabilization. One of the primary benefits of organic solvent-based binders is their ability to improve the dispersion of solid particles or nanoparticles [16]. Organic solvents reduce the surface tension between the particles, thereby promoting a more uniform distribution. This is particularly beneficial for hydrophobic or naturally poorly dispersible substances such as nanoparticles [17]. Organic solvent-based binders are highly effective in preventing the agglomeration of various components of a formulation. Using an organic solvent-based binder also prevents crystalline transitions by forming a protective layer on the particle surface, shielding the drug from external factors such as temperature, humidity, and pressure [18]. However, organic solvents require strict residual solvent management, because incomplete removal can compromise product stability and safety.

Porous carriers have emerged as viable excipient solutions for overcoming some of these challenges. Their stable pore structures, high surface areas, and tunable pore sizes provide interstitial spaces between excipients, reducing drying time and enhancing granule flowability [19,20]. The integration of porous carriers in organic solvent-based granulating liquid has been shown to improve process efficiency and formulation stability [21].

This study aimed to develop a miniaturized FEX tablet to enhance patient compliance, while maintaining bioequivalence with the marketed formulation. The formulation strategy involved the use of an organic solvent-based granulating liquid and a porous carrier to achieve a smaller tablet size, while ensuring bioequivalence with the commercial product. Furthermore, a design of experiments (DoE) approach was utilized to evaluate the effects of porous carriers and tablet size on the dissolution and residual solvent content. In addition, scale-up studies were conducted to assess the commercial feasibility of these formulations. Finally, stability, in vitro dissolution, and in vivo bioequivalence studies were performed to validate the commercial viability of the optimized formulation.

## 2. Materials and Methods

### 2.1. Materials

FEX was supplied by Ind-Swift (Punjab, Chandigarh, India). Microcrystalline cellulose 101 (MCC) was purchased from Mingtai Chemical Co. (Taoyuan City, Taiwan). FEX standard was obtained from USP (Frederick, MD, USA), and Impurity A standard was purchased from Supelco (Bellefonte, PA, USA). Low-Substituted Hydroxypropyl Cellulose (L-HPC^®^ LH-21) and hypromellose (Pharmacoat^®^ 615) were obtained from Shinetsu (Tokyo, Japan). Lactose (Pharmatose^®^ 200) was purchased from DFE pharma (Goch, Germany). Stearic acid was purchased from Sigma-Aldrich (St. Louis, MO, USA). Pregelatinized starch (PCS, Starch 1500) and Opadry TF 265F640015 (pink) were obtained from Colorcon (Indianapolis, IN, USA). Croscarmellose sodium (CMC-Na) was purchased from DuPont (Midland, MI, USA). Polyvinylpyrrolidone K30 (PVP K30) was obtained from BASF (Ludwigshafen, Germany). Magnesium stearate (Mg.st) was purchased from FACI (Carasco, Italy). Colloidal silicon dioxide (SiO_2_) with the product name Aerosil 200 was obtained from EVONIK (Rheinfelden, Germany). Ethanol (99.5%, fermentation-derived) was purchased from Daejung Chemicals & Metals Co., Ltd. (Siheung, Republic of Korea). All other reagents were of analytical or HPLC grade.

### 2.2. High-Performance Liquid Chromatography

A high-performance liquid chromatography (HPLC) system (Agilent, Santa Clara, CA, USA) was used for dissolution, content, and impurity analyses. The analytical method was performed in accordance with the United States Pharmacopoeia USP 42–NF 37 (2019) [22]. The fexofenadine reference standard and Impurity A were used in the experiments. All experiments were conducted using UV detection at 220 nm, with a correlation coefficient (*r*) exceeding 0.99. For the content analysis, a Spherisorb^®^ Phenyl column (4.6 × 250 mm, 5 µm) (Waters, Milford, MA, USA) was used with a 36:64 (*v*/*v*) mobile phase consisting of a buffer solution (prepared by dissolving acetonitrile, triethylamine, and glacial acetic acid) and acetonitrile. The flow rate was 1.5 mL/min, and the injection volume was 20 µL. For the dissolution analysis, a Spherisorb^®^ ODS2 column (4.6 × 100 mm, 5 µm; Waters) was utilized with a 30:70 (*v*/*v*) mobile phase composed of a buffer solution (prepared by dissolving sodium dihydrogen phosphate and sodium perchlorate) and acetonitrile, at a flow rate of 1.0 mL/min and an injection volume of 15 µL. For the impurity analysis, a ZORBAX^®^ SB-Phenyl column (4.6 × 250 mm, 5 µm; Agilent) was employed with the same 36:64 (*v*/*v*) mobile phase as in the content analysis, using a flow rate of 1.5 mL/min and an injection volume of 20 µL. All HPLC experiments were conducted under controlled laboratory conditions, including a temperature of 25 ± 2 °C and relative humidity of 60 ± 5%, in compliance with ICH guidelines, ensuring high precision and accuracy.

### 2.3. Compatibility Test

To determine the most suitable excipients for formulation, compatibility studies were conducted by mixing FEX with various excipients in a 1:1 ratio. The evaluated excipients included three types of diluents (microcrystalline cellulose, pregelatinized starch, and lactose monohydrate), two types of disintegrants, two types of glidants (porous materials), two types of binders (PVP K30 and hypromellose), and two lubricants (Mg.st and stearic acid). These excipients were stored in open glass vials conditions at 40 °C and 75% relative humidity to assess their stability. The impurity levels were analyzed using the validated impurity analysis method described in Section 2.2 at the initial time point as well as after two and four weeks of storage.

### 2.4. Experimental Formulation Study

**Figure 1:**
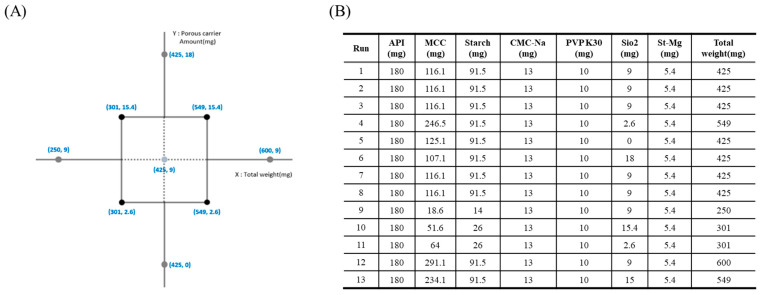
Experimental formulation using the central composite design method: (**A**) central composite design; (**B**) formulation and design matrix.

A DoE approach was implemented using Minitab^®^ ver21.4.2 (Minitab Inc., State College, PA, USA) to systematically analyze the relationships between experimental variables and responses. A central composite design (CCD) was employed to facilitate the development of an optimal formulation, as illustrated in Figure 1A. The CCD incorporates factorial, axial, and central points to ensure comprehensive exploration of the experimental space. Factorial points were employed to explore extreme values within the design space, whereas axial points extended beyond these limits to assess the potential nonlinearity in the responses. Center points were included to evaluate experimental variability and estimate potential errors. In this study, tablet weight (five levels) and amount of porous excipient (five levels) were selected as the key independent variables. For the experimental formulation study with a total weight ranging from 425 to 600 mg, the amount of MCC was adjusted. For those with a total weight of 250 to 300 mg, both MCC and pregelatinized starch contents were modified. The ratio of MCC to pregelatinized starch was determined based on prior formulation experience to achieve appropriate compressibility and tablet weight. These factors were selected to examine their influence on the dissolution rate and residual solvents, which are considered critical quality attributes (CQAs) of the final product. Thirteen experiments were conducted based on the CCD matrix to ensure adequate statistical power for the optimization. The experimental formulation and design matrix are shown in Figure 1B. FEX, MCC, PCS, CMC-Na, and SiO_2_ were placed in a P 1–6 high-speed mixer (Diosna, Osnabrück, Germany) and mixed at 250 rpm for the agitator and 2200 rpm for the chopper for 3 min while adding granulating liquid as 100% EtOH. After mixing, the granules were dried at 50 °C for 2 h, passed through a 1 mm sieve, and then mixed with Mg.st. The final granules were compressed at 10 kPa using an Autotab-200TR (Ichihashi Seiki, Kyoto, Japan).

### 2.5. Dissolution Test at pH 4.0

Dissolution testing was conducted according to the USP Apparatus 2 guidelines (Paddle Apparatus; 708-DS, Agilent) using 900 mL of pH 4.0 dissolution medium prepared with acetic acid buffer maintained at 37 ± 0.5 °C with a paddle rotation speed of 50 rpm. An aliquot of the sample (8 mL) was extracted at each sampling time, filtered through a 0.45 µm, 25 mm regenerated cellulose membrane filter (Sartorius, Göttingen, Germany), and used as the test solution. The samples were analyzed using the validated dissolution analysis method described in Section 2.2. Differences in 30 min dissolution were evaluated using one-way analysis of variance (ANOVA), followed by Dunnett’s post hoc test to compare each formulation with the reference listed drug (RLD) while controlling the Type I error rate. Statistical analyses were performed using GraphPad Prism 8 (version 8.4.2; San Diego, CA, USA). *p*-Values less than 0.05 were considered statistically significant.

### 2.6. Residual Solvents Measurement Using Gas Chromatography

To measure the residual solvent, a standard solution was prepared based on a method developed in-house by our laboratory. An ethanol standard (500 mg) was precisely weighed, placed in a 100 mL volumetric flask, and dissolved in dimethyl sulfoxide (DMSO). An aliquot of 5 mL of this solution was added to a 50 mL volumetric flask, diluted to the mark with DMSO, and 5 mL of this solution was added to a headspace vial, which was then sealed and used as the standard solution (final concentration: 500 μg/mL). The test solution was prepared by evenly crushing five tablets, precisely weighing 500 mg of this powder into a headspace vial, adding 5 mL of DMSO, mixing thoroughly, and sealing the vial for use as the test solution. The analysis was performed using gas chromatography equipment (GC; 7890 B, Agilent) equipped with a flame ionization detector. A capillary column with an internal diameter of 0.53 mm, length of 30 m, and stationary phase coating of 6% cyanopropylphenyl and 94% dimethylpolysiloxane was used. Nitrogen was used as the carrier gas at a flow rate of 3.0 mL/min and split ratio of 1:5. The injector temperature was set to 150 °C and the detector temperature to 250 °C, with airflow at 350 mL/min, hydrogen flow at 35 mL/min, and makeup gas flow at 25 mL/min. The column temperature was initially maintained at 40 °C for 10 min, then increased at a rate of 45 °C per minute to 240 °C, where it was held for 1 min. The headspace analysis conditions were set as follows: oven temperature at 80 °C, loop temperature at 90 °C, and transfer line temperature at 100 °C. The injection volume was 1 mL, the injection time was 1 min, and the GC analysis cycle was set to 30 min.

### 2.7. Formulation Study

Based on the formulation derived from the experimental design (325 mg total weight including 10 mg of porous agent), an additional formulation study was conducted using a slightly modified composition of 330 mg total weight and 9 mg of porous agent. Furthermore, to improve flowability and tabletability of the granules, the formulation components were partially divided into inside and outside phases during the study. To evaluate the dissolution rate based on the ethanol content (50%, 80%, and 100%) in the binding solvent, porous material amount, and types, samples F1–F7 were prepared according to the compositions shown in Table 1. First, PVP K30 was dissolved in ethanol solution. FEX, MCC, PCS, CMC-Na, and SiO_2_ were placed in a P 1–6 high-speed mixer (Diosna) and mixed at 100 rpm for the agitator and 2000 rpm for the chopper for 3 min. The ethanol solution was then added, and the mixture was agitated at 50 rpm and chopped at 2000 rpm for 3 min. After the ethanol solution was completely added, mixing was continued at 250 rpm for the agitator and at 2200 rpm for the chopper for 2 min. After mixing, the granules were dried at 50 °C, passed through a 1 mm sieve, and then mixed with CMC-Na, SiO_2_, and St-Mg. The final granules were compressed at 10 kPa using the Autotab-200TR (Ichihashi Seiki). The dissolution rate was studied in a medium with pH 4.0. All experiments were conducted four times.

### 2.8. Scale-Up Study

The F2 formulation was selected for further research based on the dissolution profile comparison at pH 4.0 with the RLD at 30 min. During scale-up, mixing speed and time were identified as the most critical process parameters affecting product performance. Based on the optimized formulation and manufacturing process at the laboratory scale, the scale-up was performed to a batch size of 200,000 tablets by applying the Froude number [23,24].

(1) $$F_{r}=\frac{rpm^{2}\times R}{g}$$

where rpm is the number of revolutions per minute, R denotes the agitator radius, and g denotes the gravitational constant. At the laboratory scale, a mixing speed of 250 rpm and mixing time of 2–4 min were applied. For the scale-up, the mixing speed was adjusted to 80–100 rpm, and the mixing time was set to 0.5–2 min. The final granules were compressed at 7~9 kPa using PR-2000 (PTK). The parameters for the granulation and tableting processes are listed in Table 2.

The flowability, hardness, friability, dissolution rate, and residual solvent content were evaluated. The flowability of granules was determined based on their bulk and tapped densities. Bulk and tapped densities of the granules were determined using an MT-1000 instrument (Seishin Enterprise Co., Tokyo, Japan). Bulk density was determined after dropping the powder in a 100 mL mass cylinder, and tapped density was determined after 500 taps at 250 taps per min. Each analysis was repeated thrice. The compressibility index was determined using Equation (2).

(2) $$Carr^{’}s\;Index=\frac{BD-TD}{BD}\times 100\%$$

where *BD* and *TD* are the bulk and tapped densities of the granules, respectively.

A tablet hardness tester (TBH 425 TD; Erweka GmbH, Heusenstamm, Germany) was used to measure tablet hardness. The crushing strength is recorded in kp. The mean value of ten tablets was used for each sample. Tablet friability was measured according to the USP method by weighing 20 tablets with a total weight exceeding 6.5 g before and after rotation at 25 rpm for 4 min, using a friability tester (TAR 120; Erweka). The friability was calculated using Equation (3):

(3) $$Friability=\frac{w_{1}-w_{2}}{w_{1}}\times 100\%$$

where *w*_1_ is the weight before rotation and *w*_2_ is the weight after rotation.

### 2.9. Tablet Coating

The tablets from Scale-Up 3 were coated with Opadry TF 265F640015 (pink) using an SFC-130 tablet coater (Sejeong Pharmatech, Incheon, Korea) until a weight gain of 4.5% was achieved. The coating process was conducted under the following conditions: inlet temperature at 40 °C, outlet temperature at 42–43 °C, atomizing pressure of 0.3 MPa, pump speed maintained for 20–30 min, coating pan rotation at 3–7 rpm, and spray distance of 24 cm. The dissolution tests of the coated Scale-Up 3 formulation were performed in various dissolution media, including pH 1.2, 4.0, and 6.8 buffers, maintained at 37 ± 0.5 °C with a paddle rotation speed of 50 rpm. The dissolution samples were analyzed according to the method described in Section 2.5, and the residual solvents were evaluated as outlined in Section 2.6.

### 2.10. Accelerated Stress Test

In this study, the coated Scale-Up 3 samples were stored under accelerated conditions (40 °C, 75% relative humidity) for the initial period, as well as for 3 and 6 months, to assess their stability. The samples were packaged in bottles consisting of a high-density polyethylene (HDPE) body and a low-density polyethylene (LDPE) cap, sealed with aluminum foil to ensure a closed system during storage. The assessment parameters included the assay, dissolution profile, impurity levels, and polymorphic stability. The assay and dissolution rates were measured using HPLC, allowing for a quantitative assessment of the stability of the active ingredient. Impurities were also monitored using HPLC to track the accumulation of degradants. Polymorphic changes were analyzed using powder D2 Phase X-ray diffraction (PXRD, Bruker, Billerica, MA, USA), where the variation in the 2θ values was used to determine any polymorphic transitions. PXRD patterns were recorded using a Cu Kα radiation source operating at 40 kV and 40 mA. Each sample in the X-ray holder was continuously scanned at a range of 5°/min over a 2θ range from 5° to 40°, and the result was processed using a pre-loaded program.

### 2.11. In Vivo Bioequivalence

This pharmacokinetic study was approved by the Institutional Review Board (IRB) of Bumin Hospital (Seoul, Republic of Korea; IRB File No. BMH2023-06-020). All subjects (60 participants) were enrolled in the study after completing a medical history review, physical examination, and standard laboratory tests. This study employed an open-label, randomized, fasting, single-dose, two-treatment, two-period, crossover design. The subjects consumed a test tablet, which was manufactured in a facility compliant with Good Manufacturing Practice (GMP) standards, with 150 mL of water after fasting for 10 h overnight. Blood samples were collected pre-dose (0 h), at 10, 20, 30, and 45 min, and at 1, 1.25, 1.5, 2, 3, 4, 6, 8, 12, and 24 h. Collected plasma was centrifuged at 3000 rpm for 10 min and transferred to two Eppendorf tubes per sample, then stored at −80 °C until analysis. FEX concentrations were analyzed using LC-ESI-MS/MS equipment that had been validated for selectivity, linearity, accuracy, precision, and stability in human plasma. The analytical method is summarized as follows: HPLC-ESI-MS/MS analyses were carried out on an ACQUITY UPLC system (Waters) composed of a quaternary pump, an autosampler, a column oven, and a Xevo TQ-S micro triple quadrupole mass spectrometer (Waters) equipped with an electrospray ion source. LC separation was performed on a Hypersil GOLD^TM^ C18 column (100 mm × 2.1 mm i.d.; 1.9 µm particle size; Thermo Fisher Scientific, Waltham, MA, USA) using the gradient method with mobile phase A (0.1% formic acid in 5 mM ammonium formate) and mobile phase B (0.1% formic acid in acetonitrile). The initial concentration of mobile A was set at 70%, and it was gradually reduced to 10% over the course of one minute at a flow rate of 0.35 mL/min and at 40 °C. The run time was 5 min in, and the injection volume was 1 µL. Mass spectrometric detection was performed using an electrospray ion source in positive ionization mode. The turbo-gas temperature was 600 °C, with an ion spray needle voltage of 2.5 kV. The collision energy was optimized at 35 V for fexofenadine. Multiple Reaction Monitoring (MRM) was used for data acquisition. The MRM fragmentation transitions were *m*/*z* 502.30 → 171.00.

## 3. Results and Discussion

### 3.1. Selection of Excipients

A compatibility study was conducted to evaluate the potential interactions between FEX and the excipients, and the results are presented in Table 3. Overall, most of the excipients exhibited good compatibility with the active ingredient under accelerated storage conditions, with impurities remaining below the limit of quantitation (LOQ: 0.24 μg/mL). PVP K30 showed a slight impurity formation. However, because it can be used as a binder at a concentration of less than 5%, the formulation was designed to remain consistent with products on the market. This was not expected to affect the product stability. Among the excipients evaluated for formulation compatibility, MCC, PCS, CMC-Na, PVP K30, SiO_2_, calcium silicate, and Mg.st were selected for further formulation studies.

### 3.2. Dissolution Rate and Residual Solvent of the Design Model

**Figure 2:**
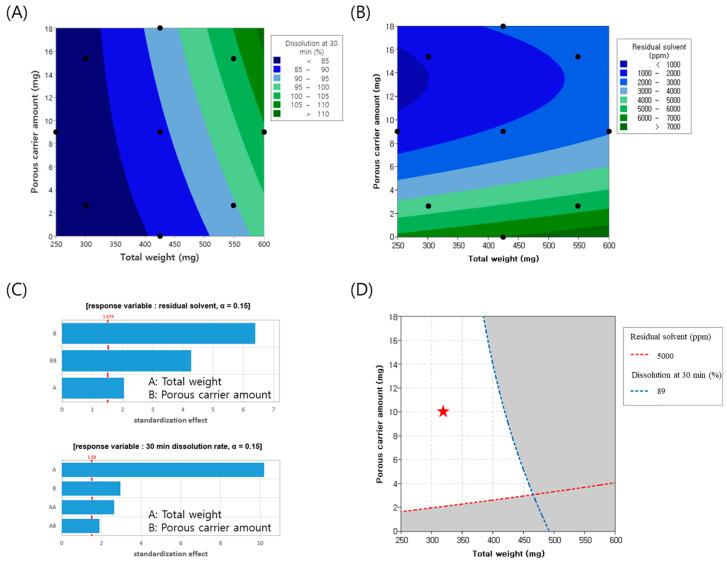
The results of modeling the design of experiments: (**A**) dissolution rate contour plot for colloidal silicon dioxide and total weight, (**B**) residual solvent contour plot for colloidal silicon dioxide and total weight, (**C**) Pareto chart, and (**D**) optimization plot showing the effect of colloidal silicon dioxide and total weight. The red star represents the optimal point.

To minimize the tablet weight, experiments were conducted by varying the tablet weight from 250 to 600 mg and adjusting the amount of porous carrier, colloidal silicon dioxide, from 0 to 18 mg. Throughout this process, the changes in the dissolution rate and residual solvents, which represent the CQAs of the finished product, were analyzed using contour plots, as illustrated in Figure 2A,B. Pareto chart analysis revealed that both colloidal silicon dioxide and tablet weight were significant factors, with a significance level set at 0.15. The model summary of the ANOVA and residual plots are presented in Supplementary Tables S1 and S2 and Figures S1 and S2, respectively. Specifically, the amount of colloidal silicon dioxide had the most substantial impact on the residual solvents, whereas the tablet weight was the primary determinant of the dissolution rate, as depicted in Figure 2C. As the tablet weight increased, the proportion of excipients relative to FEX decreased, potentially reducing the dilution effect between the FEX particles. This insufficient dilution may have resulted in a lower dissolution rate. Additionally, larger tablet sizes make solvent evaporation more difficult, leading to an increase in residual solvent levels. Conversely, as the proportion of porous carriers increased, the enhanced porosity facilitated solvent evaporation, thereby reducing the residual solvent content. Regarding the dissolution rate, SiO_2_ appeared to attach to FEX drug particles, partially reducing their cohesive force, which may have contributed to improved dissolution. Considering a residual solvent level below 5000 ppm and a target dissolution rate at 30 min of 79% (minimum) to 89% (maximum) to align with market product standards, the overlapping contour analysis identified the white area as the optimal experimental parameter. Throughout the experimental design, the porous carrier content was set to 10 mg and the tablet weight was set to 325 mg. Based on these parameters, the actual formulation was adjusted to incorporate 7 mg of the porous carrier within the granules and 2 mg outside the granules, resulting in a tablet weight of 330 mg. The experiments were conducted accordingly.

### 3.3. Formulation Study

**Figure 3:**
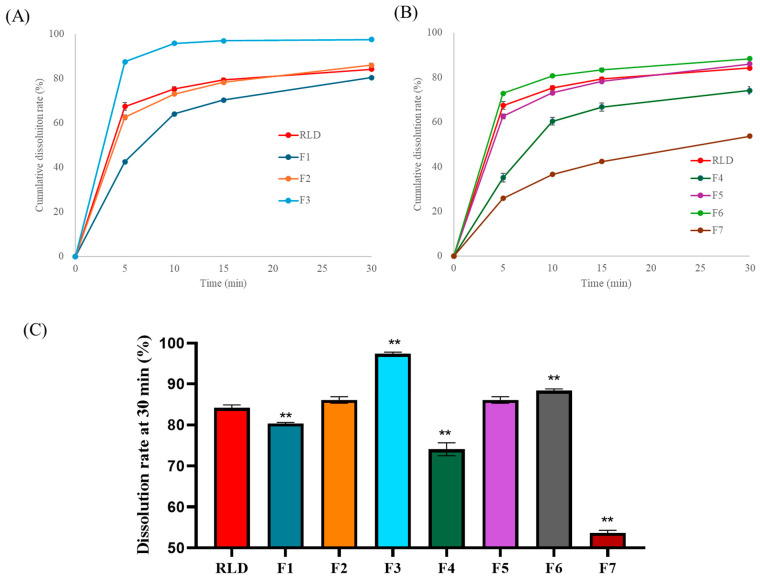
Dissolution results of RLD and F1–F7; (**A**) dissolution profiles of F1–F3 prepared using ethanol solutions of varying concentrations, (**B**) dissolution profiles of F4–F7 using different types and amounts of porous agents, and (**C**) dissolution rate at 30 min of prepared formulations (n = 4). All values are presented as mean ± standard deviation (SD). All values were statistically analyzed by one-way ANOVA with post hoc Dunnett’s test. ** *p* < 0.01; compared with RLD.

The effect of ethanol concentration in the binder solution and the type of porous material, silica dioxide, on dissolution performance was evaluated, and the results are summarized in Figure 3. The dissolution rates of F1 (50% EtOH), F2 (80% EtOH), and F3 (100% EtOH) exhibited steeper initial slopes during the first 5 min as the ethanol content increased. The percentage of drug released at 30 min was 80.4%, 86.1%, and 97.4% for F1, F2, and F3, respectively. The dissolution rate at 30 min of the marketed product was measured at 84.2%. Based on these findings, tablets prepared with an 80% ethanol solution exhibited a dissolution profile similar to that of the marketed product, leading to the selection of 80% ethanol as the optimal ethanol ratio. The improved solubility at higher ethanol concentrations was attributed to the high solubility of FEX in ethanol. During the wet granulation process, FEX dissolved in ethanol, leading to a reduction in particle size [25,26]. Consequently, this enhanced dispersion within the excipients, potentially improving the overall formulation characteristics. However, further investigation is required to confirm the extent of particle size reduction and its direct impact on formulation performance.

A comparison of the dissolution profiles of formulations containing varying amounts of silica dioxide (F4: 0/0 mg, F2: 7/2 mg, F5: 7.9/2 mg, F6: 23/2 mg) revealed that F4, which did not contain any silica, exhibited a slower dissolution rate not only during the initial phase but also at 30 min, compared to the silica-containing formulations. The percentages of drug released at 30 min were 74.1%, 86.1%, 86.1%, and 88.4% for F4, F2, F5, and F6, respectively. The dissolution rate of F4, which lacked a porous agent, was lower, whereas F6, which contained three times the amount of porous agent as F5, showed only a slight 2% increase in dissolution rate. Formulation F5 exhibited a dissolution rate comparable to F2, despite a 1 mg difference in silica dioxide content. Both F2 and F5 demonstrated dissolution behavior similar to the reference list, Allegra^®^ 180 mg, indicating that a porous agent concentration of 7/2 mg is sufficient to enhance the dispersion of FEX particles during granulation, even in miniaturized tablet formulations.

In the case of F7 (7.9/2 mg), formulated using Florite R, the 30 min dissolution rate was 53.7%, significantly lower than F5, despite having an identical porous agent concentration. This may be attributed to the differences in particle size and surface area between Florite R and silicon dioxide. While silicon dioxide has a nanoscale particle size, Florite R has a micron-sized structure, which may not sufficiently enhance the dispersion of FEX particles. Additionally, the lower dissolution rate of F7 compared to F4 suggests that the formation of silica gel via Si(OH)_4_, derived from Florite R at pH 4.0, contributed to the slower dissolution rate [27].

Table 4 presents the granule and tablet properties of F4–F7. In the case of F4, which lacked a porous agent, granules were poorly formed and exhibited agglomeration. Furthermore, after identical drying times, F4 retained more than five times the amount of residual solvent compared to F5, F6, and F7. This confirms that the addition of a porous material significantly reduces solvent drying time during granulation. Moreover, improvements were observed in granule flowability and tablet hardness, further supporting the beneficial effects of porous material incorporation in miniaturized FEX formulations.

The dissolution rates of F1 (50% EtOH), F2 (80% EtOH), and F3 (100% EtOH) were 80.4%, 86.1%, and 97.4% within 30 min, respectively. The dissolution rate of the marketed product was measured to be 84.2%. Based on these findings, tablets prepared with 80% ethanol solution demonstrated a dissolution profile similar to that of the marketed product, leading to the selection of 80% ethanol as the optimal ethanol ratio. During wet granulation, FEX dissolved in ethanol, leading to a reduction in particle size. Consequently, this may have improved dispersion within the excipients, potentially enhancing the overall formulation characteristics.

### 3.4. Characterization of Granules and Tablets Manufactured Using a Scale-Up Process

**Figure 4:**
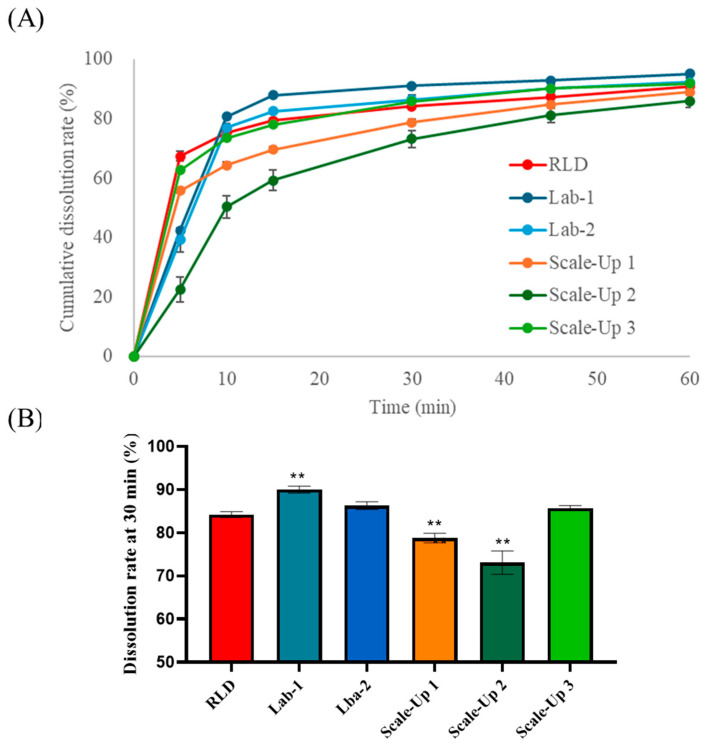
Dissolution results RLD, Lab-1 and -2, and Scale-Ups 1–3; (**A**) dissolution profiles of Lab-1 and -2 and Scale-Ups 1–3, and (**B**) dissolution rate at 30 min. All values are presented as mean ± standard deviation (SD). All values were statistically analyzed by one-way ANOVA with post hoc Dunnett’s test. ** *p* < 0.01; compared with RLD.

**Figure 5:**
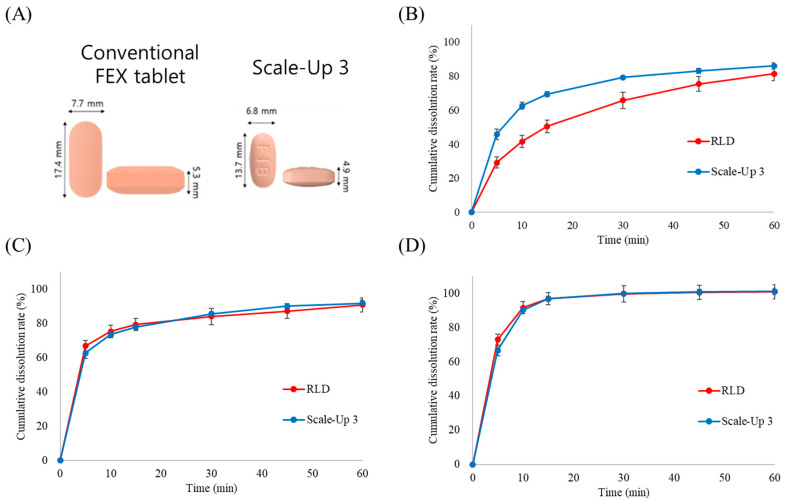
Photographs and dissolution profiles of manufactured formulation in different dissolution media: (**A**) picture of conventional fexofenadine tablets and coated Scale-Up 3 tablets, (**B**) dissolution profiles in pH 1.2, (**C**) dissolution profiles in pH 4.0, (**D**) dissolution profiles in pH 6.8 (n = 8).

This study evaluated whether the manufacturing process parameters established at the laboratory scale could maintain the performance characteristics of the formulation, particularly the dissolution rate and physical properties, during scale-up to commercial production. Initially, Lab-1 and Lab-2 batches were manufactured using the optimized laboratory-scale process with different blending times to compare the granule properties and dissolution rates. Despite a two-fold difference in blending time, no significant differences were observed in the granule properties, tablet properties, or dissolution rates between the two batches. However, the direct application of optimized laboratory conditions to a scaled-up process presented challenges. Among the various parameters, agitator speed has been identified as a critical factor influencing granule properties. Therefore, at the initial stage of the scale-up, the agitator speed was set to 100 rpm based on the Froude number derived from laboratory conditions, and the blending time was adjusted to 2 min. Various process conditions were tested to assess their impact on the granule and tablet properties. The process conditions are listed in Table 5. The bulk density (BD) of the granules varied depending on the blending conditions, showing notable differences among the scaled-up batches 1, 2, and 3. The BD of scale-up batch 3 closely resembled that of the laboratory batch. Analysis of the 30 min dissolution rate in pH 4.0 buffer demonstrated that the dissolution profile of scale-up batch 3 was highly comparable to that of both the commercial and laboratory batches. As shown in Figure 4, these results suggest that scale-up batch 3 successfully transitioned from laboratory to commercial scale. As shown in Figure 5A, the final formulation achieved a 44% reduction in weight and an approximately 50% reduction in volume compared with the marketed product, potentially improving patient compliance. A dissolution comparison between the marketed product (Allegra) and scale-up batch 3 was confirmed, as illustrated in Figure 5B–D. Dissolution testing in three different media confirmed that the dissolution profiles at pH 4.0 and pH 6.8 were nearly identical to those of the reference product. However, at pH 1.2, the initial dissolution rate was higher than that of the commercial product. This accelerated drug release at pH 1.2 is likely attributable to the enhanced solubility of FEX through porous formulation technology, despite its inherently low solubility under acidic conditions. Since the primary absorption sites for FEX are the duodenum and small intestine [28,29], the difference observed at pH 1.2 was not expected to have a significant impact on bioequivalence. An in vivo bioequivalence study in humans was conducted to evaluate the pharmacokinetic similarities between the formulations.

### 3.5. Accelerated Stress Test of Manufactured Formulation

The stability test results are summarized in Table 6. The coated Scale-Up 3 samples stored under accelerated conditions (40 °C/75% RH) for six months demonstrated consistent drug content, with 99.0% at 3 months and 99.2% at 6 months, maintaining the initial value of 99.1%. In addition, the dissolution at 30 min remained within the range of 96–99%, which is comparable to the initial dissolution range of 96–98%. The amount of related substances was consistently observed to be 0.1% throughout the storage period.

**Figure 6:**
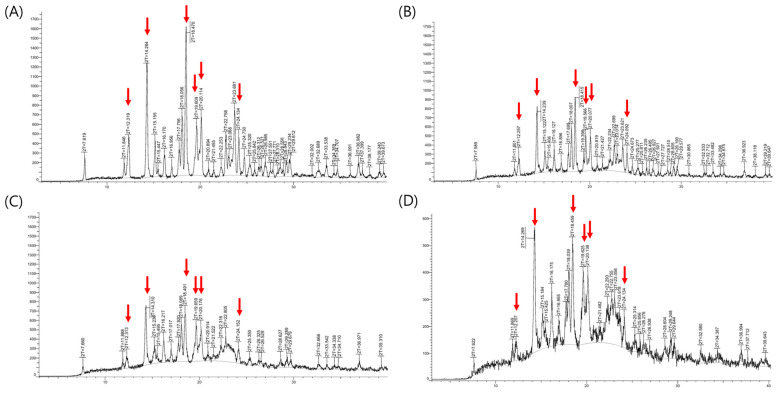
PXRD stability test results: (**A**) fexofenadine HCl; (**B**) physical mixture; (**C**) initial; (**D**) 6 months accelerated. The red arrows indicate the characteristic peaks of fexofenadine HCl form I (2-theta: 12.3°, 14.3°, 18.5°, 19.6°, 20.1°, and 24.1°).

The X-ray powder diffraction (XRPD) results were presented in Figure 6. XRPD analysis of the fexofenadine (FEX) active pharmaceutical ingredient revealed characteristic 2-theta values at 12.3°, 14.3°, 18.5°, 19.6°, 20.1°, and 24.1°, corresponding to the major peaks of FEX form I [30]. These distinctive peaks originating from the crystalline Form I were observed not only in the physical mixture and initial formulation but also after 6 months under accelerated storage conditions. No noticeable differences in peak distribution were observed between the initial and the samples stored under accelerated conditions. Although polymorphic transitions of FEX can potentially affect dissolution rates, the formulation maintained consistent drug content and dissolution performance following accelerated storage. These results indicate that the formulation retained its original crystalline form and exhibited physical stability. Furthermore, based on the ICH Q1E guideline, the demonstrated stability without significant change over 6 months under accelerated conditions (40 °C/75% RH) supports the extrapolation of a shelf life of at least two years.

### 3.6. Pharmacokinetic Assessment of Manufactured Formulation

**Figure 7:**
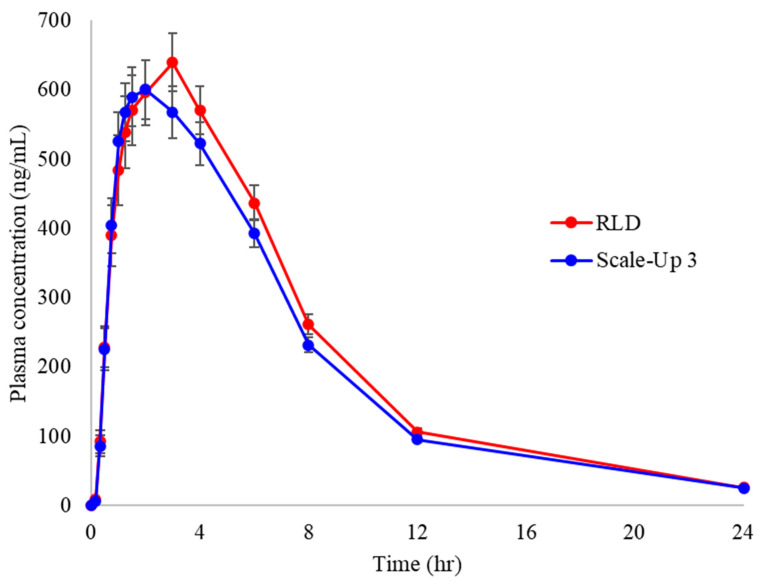
Pharmacokinetic profiles of manufactured formulation in humans (n = 60).

The approval process for generic drugs requires the demonstration of pharmaceutical properties and bioequivalence with the reference drug [31]. In this study, a pharmacokinetic evaluation was conducted to compare test and reference drugs in 60 healthy adult subjects. The plasma concentrations of both formulations were measured over a 24 h period, and key pharmacokinetic parameters, including the area under the curve (AUC), maximum plasma concentration (C_max_), time to reach maximum concentration (T_max_), and half-life (T_1/2_), were assessed. A statistical summary of the pharmacokinetic data is presented in Table 7, and the plasma concentration profiles of the test and reference drugs are shown in Figure 7. Throughout the 24 h study period, the mean plasma concentration of the test drug increased until two hours post-administration, followed by a rapid decline. Similarly, the plasma concentration profile of the reference drug exhibited a nearly identical trend, with minimal differences in the absorption and elimination patterns between the two formulations. The pharmacokinetic parameters, including AUC_0–24h_, C_max_, T_max_, and T_1/2_, remained within acceptable bioequivalence ranges, indicating that both formulations met the regulatory criteria for pharmaceutical properties and bioequivalence. The 90% confidence intervals (CIs) of the log-transformed AUC and C_max_ values were within the bioequivalence acceptance range of log 0.8 to log 1.25. Specifically, the 90% CI for the AUC ranged from log 0.8858 to log 1.0269, whereas that for the C_max_ ranged from log 0.8616 to log 1.0451. These results confirmed that the test drug exhibited pharmacokinetic characteristics comparable to those of the reference drug, meeting the bioequivalence requirements. A statistical comparison of pharmacokinetic parameters was performed using Student’s paired t-test, yielding a *p*-value greater than 0.05, which indicated no significant differences in absorption, distribution, or elimination between the two formulations. Therefore, under the conditions used in this study, the test formulation was found to be bioequivalent to the reference formulation.

## 4. Conclusions

This study focused on the development of a miniaturized and commercially viable FEX tablet. Through formulation research, a tablet was successfully developed that achieved a 44% reduction in weight and a 50% reduction in volume compared to the marketed product, while maintaining an equivalent dissolution rate at pH 4.0 and lower residual solvent content. Further process optimization enabled the establishment of a large-scale manufacturing process. Accelerated stress tests confirmed that both the dissolution profile and crystalline form remained stable, indicating high stability under commercial conditions. Dissolution testing under three different pH conditions demonstrated that the dissolution rates at pH 4.0 and pH 6.8 were comparable to those of the reference product. However, at pH 1.2, the formulation exhibited a faster dissolution rate owing to enhanced solubilization. Clinical studies confirmed that the developed formulation was bioequivalent to the marketed product. In this study, we successfully developed a miniaturized, commercially viable FEX tablet. This formulation, which results in a 44% reduction in tablet weight, enhances patient compliance, particularly for individuals who experience difficulty swallowing larger tablets, ultimately improving medication adherence. These results provide a strategic approach for overcoming formulation challenges related to size reduction, solubility, and manufacturing efficiency and offer valuable guidance for future pharmaceutical product development.

## Figures and Tables

**Table 1:** Composition of fexofenadine tablet formulations.

Component (mg/Tablet)		F1	F2	F3	F4	F5	F6	F7
Inside	Fexofenadine HCl	180	180	180	180	180	180	180
	MCC 101	67.6	67.6	67.6	76.6	66.7	51.6	66.7
	Pregelatinized starch	45	45	45	45	45	45	45
	CMC-Na	6	6	6	6	6	6	6
	PVP K30	10	10	10	10	10	10	10
	SiO_2_	7	7	7	-	7.9	23	-
	Florite R	-	-	-	-	-	-	7.9
Outside	CMC-Na	7	7	7	7	7	7	7
	SiO_2_	2	2	2	-	2	2	-
	Florite R	-	-	-	-	-	-	2
	Mg.st	5.4	5.4	5.4	5.4	5.4	5.4	5.4
Ethanol solution		50 (50%)	50 (80%)	50 (100%)	50 (80%)	50 (80%)	50 (80%)	50 (80%)
Total (mg)		330	330	330	330	330	330	330

**Table 2:** Manufacturing conditions and properties of granules and tablets in lab scale and scale-up.

Process		Lab-1	Lab-2	Scale-Up 1		Scale-Up 2	Scale-Up 3
Batch size (tablets)		500	500	200,000		200,000	200,000
Kneading	Agitator (rpm)	250	250	100		100	80
	Chopper (rpm)	2200	2200	3000		3000	3000
	Kneading time (min)	2	4	2		0.5	1.5
Drying		50 °C, 2 h			50 °C, 4 h		
Sieving		1.0 mm					
Compression (kN)		8			7~9		

**Table 3:** Compatibility testing of fexofenadine hydrochloride under accelerated stress conditions (40 °C/RH 75%).

Type	Excipient	Initial	2 Weeks	4 Weeks
Diluent	Microcrystalline cellulose 101	<LOQ	<LOQ	<LOQ
	Pregelatinized starch	<LOQ	<LOQ	<LOQ
	Lactose monohydrate	<LOQ	<LOQ	<LOQ
Disintegrant	Croscarmellose sodium (CMC-Na)	<LOQ	<LOQ	<LOQ
	Low-Substituted Hydroxypropyl Cellulose	<LOQ	<LOQ	<LOQ
Glidant (porous materials)	SiO_2_	<LOQ	<LOQ	<LOQ
	Florite R (calcium silicate)	<LOQ	<LOQ	<LOQ
Binder	Polyvinylpyrrolidone K30	<LOQ	Impurity A 0.45	Impurity A 0.84
	Hypromellose	<LOQ	<LOQ	<LOQ
Lubricant	Magnesium stearate	<LOQ	<LOQ	<LOQ
	Stearic acid	<LOQ	<LOQ	<LOQ

Limit of quantification (LOQ) of the impurity A and unknown impurity: 0.445 μg/mL and 0.240 μg/mL, respectively.

**Table 4:** Properties of granules and tablets based on the quantity and type of porous materials.

Process		F4	F5	F6	F7
Granule properties	Before drying	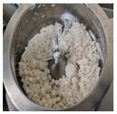	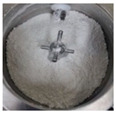	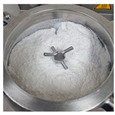	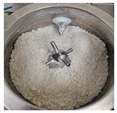
	Carr’s Index (%)	19.2 (Fair)	22.6 (Passable)	23.5 (Passable)	22.5 (Passable)
Tablet properties	Hardness (kp)	11	18	20	15
	Friability (%)	0.03	0.04	0.05	0.07
	Disintegration time (min)	<5	<2	<2	<3
	Residual solvents (ppm)	17,426	2689	2172	3380

**Table 5:** Properties of granules and tablets in lab scale and scale-up.

**Process**		**Lab-1**	**Lab-2**	**Scale-Up 1**	**Scale-Up 2**	**Scale-Up 3**
Granule properties	Carr’s Index (%)	23.39 (Passable)	21.95 (Passable)	16.36 (Fair)	24.52 (Passable)	20.09 (Fair)
Tablet properties	Hardness (kp)	14	13	6	9	11
	Friability (%)	0.06	0.08	0.07	0.03	0.05
	Disintegration time (min)	<3	<3	< 3	<3	<3
	Residual solvents (ppm)	1958	1351	1595	871	995

**Table 6:** The results of the manufactured tablet after accelerated stress test (40 °C/75% RH).

**Parameters** **(Specification)**	**Initial**	**3 Months**	**6 Months**
Assay (93~107%)	99.1%	99.0%	99.2%
Dissolution (NLT 80% in 30 min)	96~98%	96~99%	96~99%
Total impurity (NMT 0.5%)	0.1%	0.1%	0.1%

NLT: not less than, NMT: not more than.

**Table 7:** Pharmacokinetic parameters of manufactured tablet (n = 60).

Parameters	RLD	Scale-Up 3	90% CI (Acceptance Criterion: log 0.8~log 1.25)
AUC_0–24h_ (h_·_ng/mL)	5216.095 ± 2160.794	4873.704 ± 1755.350	log 0.8858~1.0269
C_max_ (ng/mL)	775.834 ± 377.448	732.556 ± 352.033	log 0.8616~1.0451
T_max_ (h)	2.04 (0.50~6.00)	2.00 (0.75~6.00)	-
T_1/2_ (h)	4.70 ± 0.73	4.88 ± 1.05	-

## Data Availability

The data presented in this study are available upon request from the corresponding author.

## Supplementary Materials

The following supporting information can be downloaded at https://www.mdpi.com/article/10.3390/pharmaceutics17060756/s1. Table S1. Summary of ANOVA results (with a significance level set at 0.15) for the effects of total weight and porous carrier amount on residual solvent (ppm); Table S2. Summary of ANOVA results (with a significance level set at 0.15) for the effects of total weight and porous carrier amount on dissolution at 30 min (%); Figure S1. Residual plots for residual solvent (ppm); (A) Normal probability plot, (B) Versus Fits, (C) Histogram, and (D) Versus order; Figure S2. Residual plots for dissolution at 30 min (%); (A) Normal probability plot, (B) Versus Fits, (C) Histogram, and (D) Versus order.

## Abbreviations

The following abbreviations are used in this manuscript:

FEX	Fexofenadine
RH	Relative humidity
API	Active pharmaceutical ingredient
MCC	Microcrystalline cellulose
PCS	Pregelatinized starch
CMC-NA	Croscarmellose sodium
PVP K30	Polyvinylpyrrolidone K30
Mg.st	Magnesium stearate
SiO_2_	Silicon dioxide
HPLC	High-performance liquid chromatography
DoE	Design of experiments
CCD	Central composite design
CQA	Critical quality attributes
GC	Gas chromatography
BD	Bulk density
TD	Tapped density
AUC	Area under the curve
C_max_	Maximum plasma concentration
T_max_	Time to reach maximum concentration
T_1/2_	Half-life
CI	Confidence interval
