# Supplementary material for: Formulation and Bioequivalence Evaluation of a Miniaturized Fexofenadine Hydrochloride Tablet
Source: Pharmaceutics. 2025 Jun 8;17(6):756. doi: 10.3390/pharmaceutics17060756 (PMC12196470; doi:10.3390/pharmaceutics17060756)
Supplement: Supplementary file 1 [file pharmaceutics-17-00756-s001.zip › pharmaceutics-3563022-supplementary.pdf]

Supplementary data

Supplementary Table S1. Summary of ANOVA results (with a significance level set at 0.15) for the effects of total weight and porous carrier amount on residual solvent (ppm)

**Source**	**DF**	**Adj SS**	**Adj MS**	**F-Value**	**P-Value**
Model	3	36393097	12131032	21.26	0.000
Linear	2	25899558	12949779	22.69	0.000
Total weight (mg)	1	2423050	2423050	4.25	0.069
Porous carrier amount (mg)	1	23476508	23476508	41.13	0.000
Square	1	10493539	10493539	18.39	0.002
Porous carrier amount (mg)*Porous carrier amount (mg)	1	10493539	10493539	18.39	0.002
Error	9	5136497	570722		
Lack-of-Fit	5	5124217	1024843	333.80	0.000
Pure Error	4	12281	3070		
Total	12	41529595			

Supplementary Table S2. Summary of ANOVA results (with a significance level set at 0.15) for the effects of total weight and porous carrier amount on dissolution at 30 min (%)

Source	DF	Adj SS	Adj MS	F-Value	P-Value
Model	4	579.688	144.922	30.89	0.000
Linear	2	529.016	264.508	56.39	0.000
Total weight (mg)	1	486.462	486.462	103.70	0.000
Porous carrier amount (mg)	1	42.554	42.554	9.07	0.017
Square	1	33.532	33.532	7.15	0.028
Total weight (mg)*Total weight (mg)	1	33.532	33.532	7.15	0.028
2-Way Interaction	1	17.140	17.140	3.65	0.092
Total weight (mg)*Porous carrier amount (mg)	1	17.140	17.140	3.65	0.092
Error	8	37.529	4.691		
Lack-of-Fit	4	36.670	9.167	42.69	0.002
Pure Error	4	0.859	0.215		
Total	12	617.216			

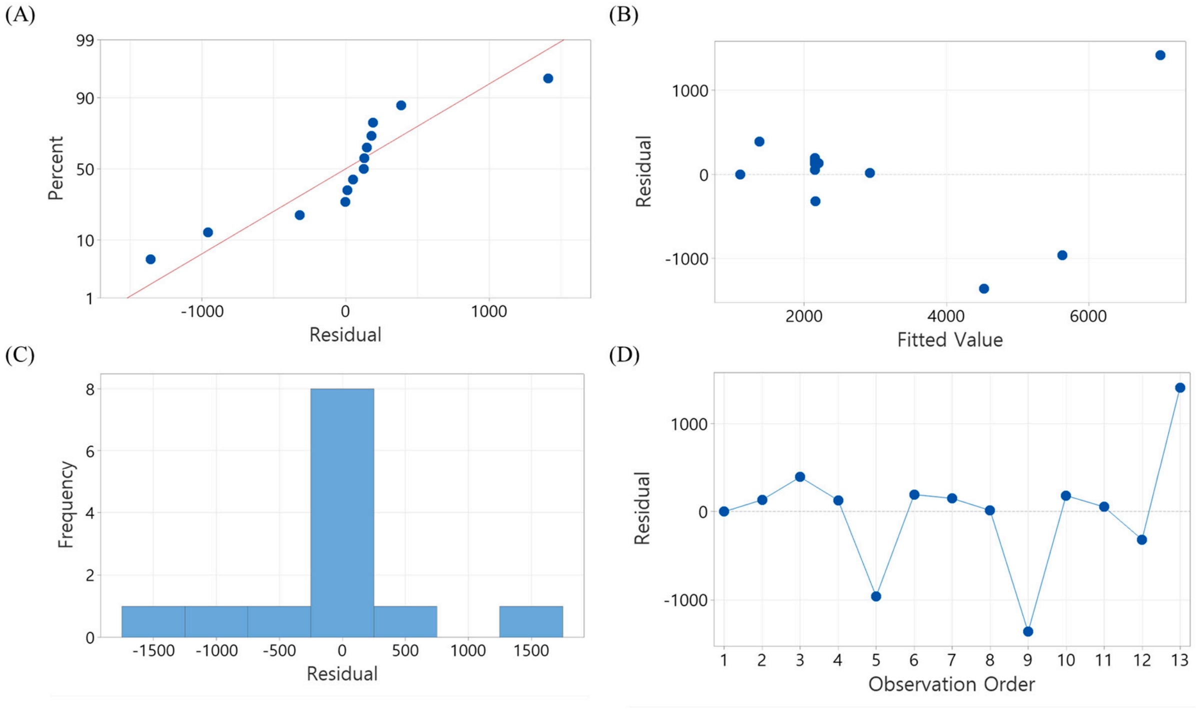

Supplementary Figure S1. Residual plots for residual solvent (ppm); (A) Normal probability plot, (B) Versus Fits, (C) Histogram, and (D) Versus order.

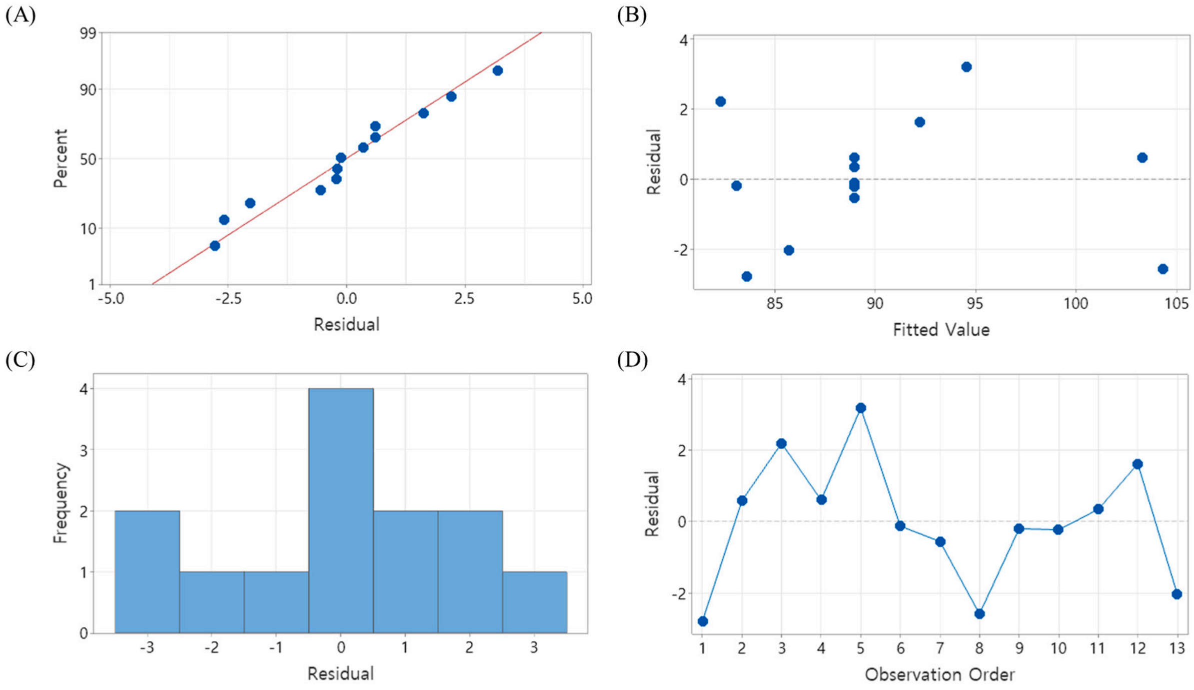

Supplementary Figure S2. Residual plots for dissolution at 30 min (%); (A) Normal probability plot, (B) Versus Fits, (C) Histogram, and (D) Versus order.
